# Human Schistosome Infection and Allergic Sensitisation

**DOI:** 10.1155/2012/154743

**Published:** 2012-08-27

**Authors:** Nadine Rujeni, David W. Taylor, Francisca Mutapi

**Affiliations:** Institute of Immunology and Infection Research, Centre for Immunity, Infection, and Evolution, School of Biological Sciences, University of Edinburgh, Ashworth Laboratories, King's Buildings, West Mains Rd, Edinburgh EH9 3JT, UK

## Abstract

Several field studies have reported an inverse relationship between the prevalence of helminth infections and that of allergic sensitisation/atopy. Recent studies show that immune responses induced by helminth parasites are, to an extent, comparable to allergic sensitisation. However, helminth products induce regulatory responses capable of inhibiting not only antiparasite immune responses, but also allergic sensitisation. The relative effects of this immunomodulation on the development of protective schistosome-specific responses in humans has yet to be demonstrated at population level, and the clinical significance of immunomodulation of allergic disease is still controversial. Nonetheless, similarities in immune responses against helminths and allergens pose interesting mechanistic and evolutionary questions. This paper examines the epidemiology, biology and immunology of allergic sensitisation/atopy, and schistosome infection in human populations.

## 1. Introduction

The major human helminth parasites belong to two phyla, the nematodes (or roundworms) which include intestinal soil transmitted helminths (STH) and filarial worms (which cause lymphatic filariasis and onchocerciasis), and the platyhelminths (or flatworms) which include the flukes (or trematodes, including schistosomes) and the tapeworms (or cestodes). Although common in most parts of the world sixty years ago [1], these parasites are currently mainly prevalent in sub-Saharan Africa, Asia, and South America [2–4], where they are responsible for considerable disabilities including blindness and elephantiasis (filarial worms). Furthermore, helminth infections are responsible for morbidities that include anaemia, stunted growth, poor cognitive development, and malnutrition [5–7], hence exert a negative socioeconomic impact in some of the poorest communities in the world.

Immune-mediated diseases including auto-immune diseases (such as type 1 diabetes, inflammatory bowel diseases, and rheumatoid arthritis) and allergic diseases (such as asthma, allergic rhinitis, and atopic eczema) are reported to be more prevalent in developed countries and in urban areas of developing countries [8, 9]. But studies from Africa are demonstrating that allergic diseases are common, if not acknowledged, clinical problems in this region [10]. Immune disorders have been responsible for increased mortality and morbidity worldwide [11–13] and they negatively impact on economic growth due to their elevated cost of their treatment [14, 15]. There is also mounting evidence that allergic disorders, especially allergic rhinitis, are associated with attention deficit disorder and hyperactivity in children [16, 17].

Increasing rates of childhood allergies have long been a puzzle to epidemiologists [18, 19]. Thus, studying cohorts of children born in 1946, 1958, and 1970, concluded that a “new environmental agent,” contained in breast milk and possibly infants' food was responsible for the increase in eczema. Emmanuel, reviewing medical literature published from 1820 to 1900, suggested that the hay fever “epidemic” was associated with the rapid industrial growth of the 19th century since this disorder was rarely described prior that period [19]. It was Strachan who in 1989, observing that the rate of hay fever and eczema was consistently negatively associated with family size and birth position in households, hypothesized that reduced exposure to childhood infections due to increased hygiene was responsible for the allergy epidemics. This hypothesis, currently referred to as the “hygiene hypothesis,” was subsequently supported by some epidemiological studies [20, 21] but contradicted by others [22, 23] (see summary in Table 1). In a retrospective case control study on Italian military cadets, Matricardi and colleagues were able to show that cumulative exposures to foodborne and oral-faecal infections, but not infections transmitted via other routes, were associated with a reduced risk of being atopic [24]. They suggested that the mode of transmission of the pathogen was a determining factor in subsequent protection (or lack of protection) against atopy and asthma, hence explaining inconsistencies in previous studies.

## 2. Global Burden of Schistosomiasis and Atopy

### 2.1. Schistosomiasis

Schistosomiasis accounts for up to 70 million DALYs annually [6], with an estimated 15,000 deaths [4], and children carry the heaviest burden of infection [47]. With these figures, schistosomiasis is classified second only to malaria in terms of human morbidity and mortality due to parasitic diseases [48]. Schistosomiasis is caused by infection with blood-dwelling trematodes of the genus *Schistosoma*, of which *S. haematobium, S. mansoni,* and *S. japonicum* are the main human schistosomes [49]. It is typically prevalent in rural areas where natural streams, ponds, rivers, and lakes harbouring the infected intermediate host snails, are the main sources of water for domestic or occupational purposes such as washing and fishing. School children usually become infected during swimming or collecting water, while younger children and infants become infected when accompanying adults (washing clothes or collecting water) or by being bathed in these water sources [50].

### 2.2. Atopy

Rising rates of atopic diseases have been reported in developed countries since the end of World War II [18] and currently constitute a major public health issue [51]. Demographic data in the US have shown an average increase in childhood asthma prevalence of 4.3% per year from 1980 to 1996, with associated deaths and hospitalisation increasing by 3.4% and 1.4%, respectively [52]. In the United Kingdom, according to the British Allergy Foundation, 1 in 3 people suffer from allergy at some time in their lives. This report indicates that 58% of allergic sensitisations are triggered by house dust mites (HDM), a known risk factor for developing asthma and allergic rhinitis [53, 54]. Increasing prevalence of asthma in adults over a period of 10 years and doubling in school children over 20 years have been reported in Australia [55]. A recent study involving 12 European countries and 19 centres reported incidences of asthma between 5 and 17% (average 8%), while allergic rhinitis varies between 23 and 44%, with an average of 30% [56].

In less affluent countries, comparable rates of atopic diseases are generally reported in urban and suburban areas. Thus, a prevalence of asthma of 9% was reported in urban areas of Rwanda [57] while the International Study of Asthma and Allergies in childhood (ISAAC) reported an overall prevalence of 10.9% across 22 centres in Africa [58]. Reported incidences of allergic rhinitis range from 14% to 54% in urban and suburban areas across African countries (reviewed by [59]). Importantly, according to the ISAAC phase three, although the prevalence was generally lower, there were more severe symptoms of rhinoconjnnctivitis reported in urban centres of developing countries compared to those reported in developed countries [8]. However, studies from Africa suggest that allergic conditions may be underdiagnosed in Africa due to “inappropriate” diagnostic tests and these studies call for component-resolved allergy testing in Africa [60]. Indeed a recent study in Zimbabwe showed that schistosome-infection resulted in impaired diagnosis of cat allergy [61].

## 3. Atopy and Schistosome Life-Cycle Stages

In schistosome infection, the human immune system is exposed to schistosome larvae (cercariae and schistosomula), adult worm, and egg antigens. Animal studies as well as *in vitro* studies have demonstrated immunological changes and regulatory mechanisms associated with these different life-cycle stages. The surface of cercariae (enriched in carbohydrates [62]) and the newly transformed schistosomula activate the complement cascade [63, 64] and eliciting proinflammatory responses [65, 66]. An excessive immunological reaction to skin stage cercarial antigens results in cercarial dermatitis or swimmer's itch [67], an allergic condition also occurring in contact with nonhuman schistosomes that is prevalent in developed countries [68–70]. This inflammatory reaction is rarely reported in populations in which schistosomiasis is endemic, possibly due to regulatory responses resulting from multiple exposures as has been demonstrated in mice [71]. Such regulatory responses may be induced by skin-stage schistosomula-derived molecules such as prostaglandin E_2 _(PGE_2_) which upregulates IL-10 production during skin penetration by the parasite [72]. The PGE_2_ is also secreted by the lung-stage schistosomula during migration through the capillary beds of the lungs, and this is thought to diminish eosinophil infiltrates around the parasites (and thus inflammation [73]). In addition, these parasites are capable of inhibiting the expression of endothelial adhesion molecules such as E-selectin and VCAM-1, limiting leucocyte recruitment in the lungs [74]. These anti-inflammatory mechanisms in the lungs have been suggested as potential explanations for reduced severity of asthma symptoms in schistosome-infected asthmatic patients [45], although there are no mechanistic studies from human populations to support this.

Schistosome eggs are major Th2 triggers as demonstrated in murine studies [75, 76], and they induce formation of fibrotic lesions or granulomas [77–79]. Indeed, an *S. mansoni* egg-secreted glycoprotein, omega-1, has recently been identified that conditions dendritic cells for Th2 polarisation [25]. However, egg secretions are capable of inhibiting the specific binding of chemokines such as CXCL8 (IL-8) and CCL3 (MIP-1*α*), therefore blocking chemokine-elicited migration of neutrophils and macrophages respectively during granuloma formation [80].

Schistosome adult worm antigens also induce Th2 responses and IgE in mice [81], baboons [82], and humans [83]. However, this parasite life stage elicits high levels of modulatory responses capable of inhibiting antiparasite [84] as well as allergic reactions [85]. The latter study demonstrated that worm infection induces IL-10, producing B cells that could protect mice against anaphylaxis. They later demonstrated that egg-laying worms exacerbate while single sex worms (precluding egg production) inhibit airway hyperresponsiveness [86].

Together, these studies show that the different parasite life-cycle stages are associated with different mechanisms of regulation and inflammation. Although concurrent exposure to all or most antigens is likely in endemic areas, and despite the fact that they may induce cross-reactive immune responses [87], the different parasite life-cycle stages may differentially affect atopic responses.

## 4. Epidemiology

### 4.1. Schistosomiasis

The epidemiological patterns of schistosomiasis differ from those of atopy, mainly because of their aetiology. Indeed, while schistosome infection is acquired as a result of exposure to parasites, atopy is a genetic predisposition (although the clinical manifestations are influenced by environmental factors). In schistosome endemic areas, infection levels follow a convex shape with host age, where infection intensity rises to peak in childhood-adolescence and decline in adulthood [88]. This peak was initially interpreted as arising from different water contact levels between age groups [89]. However, longitudinal studies showed that with the same exposure rate, “resistant” individuals were older than “susceptible” individuals [90–92], suggesting an age-dependent acquired resistance to reinfection. In addition, in communities of different parasite transmission, infection intensity peaks at a younger age in areas of high transmission compared to low-transmission areas [88, 93–95], a phenomenon referred to as a “peak shift.” This phenomenon has been interpreted as reflecting different rates of development of acquired resistance to infection during schistosome infection as has been reported for *Plasmodium *infections (which cause malaria) [96, 97]. This interpretation is supported by age-related changes in immune responses, with the peak of antibodies and cytokine associated with protection coinciding with the decline in infection levels have been reported in *S. haematobium* endemic areas [83, 98, 99]. More recently, Black and colleagues observed that the rate of acquisition of antischistosome protective responses by adults occupationally exposed to *S. mansoni*, following treatment, is dependent on their history of exposure, being faster in those with a longer history [100]. This study, consistent with earlier studies [101, 102], demonstrated that resistance to schistosome infection/reinfection is acquired independent of age related physiological changes [103].

### 4.2. Atopic Diseases

Atopy is the genetic predisposition to become excessively sensitised and produce high levels of IgE [104]. However, atopic diseases result from a genetic predisposition in combination with environmental stimuli such as allergens, smoke, diet, and/or infectious agents [105, 106]. The epidemiology of atopic diseases is complex as some diseases may become more prominent with age while others diminish or disappear [107]. The earliest phases of atopic diseases usually manifest during the first five years of life and the severity (and prevalence) of clinical symptoms seem to increase in late childhood/adolescence and plateau throughout adulthood [108–110] or decline for some conditions [111, 112].

It has been suggested that a natural history of allergy manifestations in atopic individuals involves progression from atopic eczema (below one year of age) to asthma or allergic rhinitis (late childhood/adolescence), a phenomenon referred to as the “atopic/allergic march” [112]. However, this is not always consistent as some children may develop atopic dermatitis long after the onset of asthma [113], while some atopic individuals may only develop one of these conditions throughout life. Longitudinal studies indicate that atopy in infancy predicts the occurrence and severity of asthma [114] and bronchial hyperresponsiveness [115] in later life. Total and allergen-specific IgE levels also seem to increase throughout childhood in allergic individuals [116]. However, a number of events occurring in the first few years of life and *in utero* are likely to influence the onset and persistence of disease. Thus, Klinnert and colleagues have shown that respiratory infections during the first year of life and parenting difficulties (e.g., postnatal maternal depression) were independent predictors of the onset of asthma during early (3 years) and late (6–8 years) childhood in children at risk [114, 117].

Microbial exposures and diet of pregnant mothers may also alter early gene expression in neonates, influencing the onset of allergy in childhood (see [106, 118]). Consistent with this hypothesis is the finding that maternal exposure to farm milk and farm animals during pregnancy was associated with demethylation within the *FOXP3* (Treg transcription factor) locus in cord blood and subsequent elevated levels of regulatory T cells (Tregs) (and their suppressive activity) in offspring [119]. Murine studies have also shown that, when exposed to a methyl rich diet during pregnancy (as may be the case for folate supplementation in humans), foetal DNA may undergo changes in methylation that results in decreased gene transcription activity, leading to subsequent enhanced development and severity of allergic diseases [120]. The study also showed that this diet-associated allergic phenotype was transgenerationally inheritable (persistence of high levels of IgE and eosinophilia into the F2 generation).

## 5. Effector Responses in Atopy and Schistosome Infection

### 5.1. Immunoglobulin E

Identified in the 1960s as a “carrier of reaginic activity” [121, 122], IgE is well known as a central player in atopic diseases and anaphylactic reactions. This antibody is part of a protein network involving its 3 receptors, namely, the Fc*ε*RI, the CD23 (or Fc*ε*RII) and galectin-3 [32], all of which can be found in soluble forms [123, 124]. The Fc*ε*RI (also termed high-affinity receptor) is mainly expressed on mast cells and basophils but also on epidermal Langerhans cells [125] and eosinophils [126, 127]. Cross-linking of these high-affinity receptors by IgE induces activation of mast cells and basophils and their degranulation. The galectin-3 receptor is expressed on neutrophils and on trophoblast cells in placentas [128], where it is thought to facilitate IgE transport [129]. The CD23 receptor facilitates the transport of IgE-antigen complexes but is also involved in the regulation of IgE synthesis [32]. Highly conserved in mammalian lineages [130], IgE is thought to have evolved as a first line of defence against helminth parasites.

IgE antibodies are naturally strongly regulated and have the lowest concentrations of all antibodies in serum of healthy nonatopic individuals [32]. Mechanisms of regulation of IgE include its short half-life in serum (12 h for murine monoclonal antibodies [131]), the poor processing of mRNA for the membrane *ε* heavy chain [132], and the negative feedback regulation by the CD23 [133]. The latter has been a subject of investigations in terms of therapeutic application in atopic diseases but also in autoimmune diseases [134] and chronic lymphocytic leukaemia [135].

### 5.2. CD23

CD23 is the low-affinity receptor for IgE and differs from the high-affinity Fc*ε*RI receptor in structure and function. Thus, while cross-linking of the latter results in degranulation of mast cells and release of mediators, engagement of membrane-bound CD23 suppresses the production of IgE by B lymphocytes [33]. CD23 has long been proposed as a natural regulator for IgE synthesis [133] although elevated levels of CD23+ B cells have been reported in atopic patients [136]. As initially suggested by Aubry and colleagues [137], CD23 not only binds IgE but also CD21, a cell-surface protein expressed on T-cell, B-cell, and follicular dendritic cells, classically identified as a receptor for complement proteins [138] or Epstein-Barr virus [137]. The interaction between CD23, IgE, and CD21 may lead to either negative or positive regulation of IgE synthesis (reviewed in [34–36]). The binding of IgE stabilises membrane-bound CD23 and inhibits IgE synthesis from activated B cells, while in the absence of IgE binding, CD23 is cleaved by ADAM10 (a disintegrin and metalloprotease protein 10), and this destabilisation enhances IgE synthesis [32]. Soluble CD23 (sCD23) fragments resulting from the cleavage can bind to IgE with different affinities and outcomes for IgE synthesis depending on their oligomerization state. Trimers bind IgE with high affinity and enhance IgE synthesis by their ability to also bind the CD21 receptor while monomers bind with low affinity but do not bind CD21 and hence inhibit IgE synthesis [36, 139].

### 5.3. Immunoglobulin 4

Serum IgG4 antibodies, the least abundant among human IgG subclasses, have long been associated with IgE-mediated diseases [140–142]. However, rather than the cause of disease, these antibodies seem to be involved in the regulation of IgE-induced anaphylactic reactions [143]. IgG4 may interfere with antigen recognition by IgE due to their similar antigenic specificity [144], although different epitope-binding [145]. In a process that involves exchange of *fab* molecules, IgG4 are structurally hetero-bivalent (each heavy chain and light chain recognising a different epitope within a single IgG4 molecule) and often function as monovalent [141, 146], to bring about anti-inflammatory effects [147]. The interaction between IgG4 and a given antigen results in small and non-pathological immune complexes (since these antibodies cannot cross-link antigens) [146]. Furthermore, in contrast to other IgG subclasses, IgG4 cannot fix complement but inhibits complement activation by IgG1 [148]. IgG4 antibodies, in allergy or helminth infection, are secreted in response to high antigen loads [141, 149, 150] but levels of the antibodies are differentially regulated by the same cytokines [151] as those regulating IgE, suggesting an important homeostatic mechanism for controlling IgE-mediated responses.

## 6. Control of Effector Responses in Atopy and Schistosome Infection

In addition to the cross-regulation between Th1 and Th2 [152] (and potentially other T cell subsets), there is growing evidence that Th2 cells interact with a complex network of other T cell subsets as well as B cells and antibodies, naturally or during disease (atopic or infectious). Thus, it has emerged that Tregs play an important role in the tolerance of ubiquitous antigens and that alterations in Treg function [153, 154] and/or the fine balance between Tregs and Th2 cells [155, 156] determines the clinical manifestation of atopy. Indeed, in healthy (nonatopic) individuals T cell polarization occurs in contact with environmental allergens but higher levels of Tregs dampen the effect of Th2 cells, leading to peripheral tolerance [156]. Tregs modulate the activity of Th2 (and Th1) cells via several mechanisms including the secretion of anti-inflammatory cytokines such as IL-10 and TGF-*β* [155, 157]. As the description and role of other recently identified T-helper cells is clarified (e.g., Th17 cells shown to be important in nonatopic asthma) regulation of Th2 mediated responses will also become clearer [158]. The role of cells such as the T-helper cells recently shown to produce both IL-17 and Th2 cytokines (IL-4, IL-5, IL-9, and IL-13) [159] in pathogenesis is currently under intense investigation. Our own group has recently described a role for Th17 responses in human schistosome-acquired immunity (submitted).

IL-10 producing B cells (Bregs) are also involved in the recruitment of Tregs, hence contributing to the regulation of Th2 responses as demonstrated in murine models of helminth infection (see [31]). IL-10 can inhibit effector functions of mast cells and eosinophils, and regulate the growth of several cells including B cells, NK cells, mast cells, and dendritic cells. Furthermore, IL-10 modulates IgE : IgG4 ratios [154] possibly by indirectly inducing the antibody switch to IgG4 in the B-cell progeny while preventing IgE production [160].

IgG4 may control IgE-mediated histamine release as has been demonstrated in filarial infection [143]. Furthermore, it has recently been shown that the binding patterns of IgG4 antibodies correspond to natural recovery from childhood IgE-mediated milk allergy [161], suggesting their potential protective role in atopic diseases, although this is still controversial [141]. In addition, early observations that IgG4 antibodies were highly elevated in sera of patients receiving allergen immunotherapy [162] have prompted the use of IgE : IgG4 ratio as a marker for successful immunotherapy [142, 163, 164]. In helminth infections, high IgG4 : IgE ratio has been associated with reduced pathology while favouring a heavy worm load [150, 165, 166]. Interestingly, IgG4 may be one of the “regulatory antibodies” resulting from IgG syalilation involved in the control of immune disorders [167, 168].

## 7. Immune Responses in Atopy

The human immune system must distinguish between a dangerous pathogen and ubiquitous environmental allergens and has evolved to mount appropriate defensive responses to the first while tolerating (or ignoring) the latter. However, a certain proportion of individuals fail to tolerate environmental allergens and develop allergic diseases such as asthma, atopic dermatitis and allergic rhinitis. These result from excessive sensitisation to ordinary exposures to allergens [104]. IgE antibodies are critical effector molecules in the pathogenesis of these diseases [169]. Mast cells and basophils are coated with specific IgE antibodies and this results in immediate hypersensitivity (release of mediators) and/or late-phase inflammatory reaction (cytokine secretion and recruitment of leucocytes).

**Figure 1:**
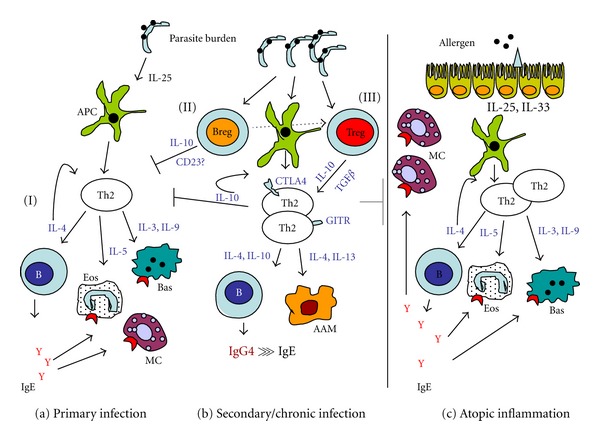
Possible regulatory mechanisms in helminth infections. Primary response (a) to parasite antigens involves Th2 polarization, IgE production, and eosinophil, mast cell, and basophil activation (I), mechanisms similar to those observed in allergic sensitisation (c). This Th2-response may be induced by parasite-secreted antigens such as the Omega-1 secreted by *S. mansoni* eggs [25]. However, with increasing parasite load or chronic infection (b), regulatory B cells are activated which suppress Th2 responses (II) via IL-10 secretion or CD23 expression [26], and/or contribute to the recruitment of Tregs [27]. Tregs (III), which may also be induced and expanded by parasite antigens [28, 29], either induce anergic Th2 cells (expressing GITR and CTLA4) unable to progress through to effector cells, or modify downstream effector functions such as B cell switch to IgG4 and/or alternative activation of macrophages, resulting in immunological tolerance (reviewed by [30]). This immunosuppression is induced in the context of helminth infection, but may also expand to allergen-induced inflammation (gray line), hence suppressing allergy. DC: dendritic cell; B: B cell, Eos: eosinophil; Bas: basophil; MC: mast cell; GITR: glucocorticoid-induced TNF*α*-related protein; CTLA4: cytotoxic T lymphocyte antigen 4; AAM: alternatively activated macrophage; Breg: regulatory B cell; Treg: regulatory T cell. The question mark (?) denotes lack of strong evidence. Figure adapted from [30, 31] and collated information from the cited references.

In atopic individuals, allergen products (e.g., cysteine proteases) activate epithelial cells, which produce thymic stromal lymphopoietin (TSLP), IL-25, and IL-33 which in turn initiate Th2 polarisation with increased production of IL-4, IL-5, IL-9, and IL-13 cytokines [170, 171]. Th2 cytokines are involved in the class-switching to IgE as well as the development and recruitment of basophils, mast cells, and eosinophils (see Figure 1(c)). IgE binds to the high-affinity Fc*ε*RI receptor on mast cells, basophils, and eosinophils which (upon exposure to allergens) results in their activation and degranulation (via cross-linking of allergens), with the release of preformed mediators such as histamine, cysteinyl leukotrienes, and prostaglandin D_2_ [154, 172].

A complex interplay between innate and adaptive immune responses underlies the heterogeneous characteristics of atopic diseases. Thus, recruitment of eosinophils into the lungs of asthmatics may be promoted not just by Th2 (IL-5) alone but in conjunction with natural killer T cells (NKT) as well as CD8+ T cells (see [173]). In addition, IL-17-producing T cells (Th17 [174]) may be involved in the severity of asthma [175]. These promote the recruitment and activation of neutrophils and lead to corticosteroids—resistant asthma [175]. IL-9-producing T-cell subset (Th9), which probably derive from Th2 cells under the influence of TGF*β*1 [176], may also be involved in the production of IgE and mast cell recruitment in the lungs [173]. In allergic rhinitis, mast cells accumulate in the epithelium of the nasal mucosa where they secrete inflammatory cytokines (IL-6, IL-8, and TNF*α*) in addition to Th2 cytokines [177].

## 8. Immune Responses during Schistosome Infection

Acquired immunity to schistosome infection was first proposed by Fisher in the 1930s when analysing data from animal studies as well as those from hospital-diagnosed *S. haematobium *infected people [88]. Subsequently, the susceptibility of schistosome larvae to immune attack was demonstrated by in vitro studies showing that sera from *S. mansoni* infected individuals could damage schistosomula in the presence of normal human peripheral blood leucocytes [178]. This “antibody-dependent” killing was subsequently shown to be eosinophil mediated [178, 179], and studies on monoclonal antibodies led to the identification of IgE antibodies with the highest cytotoxicity for the schistosomula [180–182]. Field studies were conducted to identify antibody responses predictive of resistance to reinfection following chemotherapy. Hagan et al. [83] demonstrated in a multivariate logistic regression that reinfection with *S. haematobium* was less likely in individuals producing high IgE levels against the worm antigens and more likely in those producing high levels of IgG4 against the worm or egg antigens. The role of IgE in resistance was also demonstrated by Rihet et al. [183], who identified specific antigens (120–165 KDa and 85 KDa) to which IgE reacted (on immunoblots) and showed that these antibodies, in contrast to IgM and IgG, were significantly higher in the sera of the most resistant individuals. This study showed that some of the immunogenic antigens were readily accessible to IgE on living *S. mansoni* larvae as they were located on the outer membrane. However, Dunne and colleagues, working on *S. mansoni* as well, showed that IgE (produced following treatment) against adult worm antigens, particularly a 22 kDa tegumental antigen (Sm22), but not against any other life-cycle stages, were associated with resistance to reinfection following treatment [184]. Both antiadult worm and anti-schistosomula tegument IgE antibodies were associated with resistance to *S. mansoni* reinfection in another study in Brazil [185] while antiegg IgE antibodies also could confer protection against *S. japonicum* reinfection [186].

Collectively, these studies and several others [187–190] have led to the conclusion that resistance to schistosome infection/reinfection is dependent on IgE antibodies. However, data on other antibody isotypes have been reported which correlated with resistance to infection/reinfection. For example, IgG3 against the recombinant antigen Sh13 has been associated with resistance to *S. haematobium* infection [191], while antiworm and cercariae IgM were significantly higher in individuals more resistant to reinfection with *S. mansoni* [185]. Furthermore, a decline in IgA together with an increase in IgG1 were associated with resistance acquired with host age as well as following treatment in *S. haematobium* endemic area [192]. IgA against Sm28GST antigen has also been associated with reduced *S. mansoni* fecundity and increased host resistance to reinfection [193]. More recently, antiworm IgE antibodies, as well as eosinophilia and the low affinity receptor for IgE (the CD23) have been shown to correlate with resistance in individuals undergoing multiple rounds of treatment [100, 194], again suggesting that IgE may be directly involved in parasite killing via antibody-dependent cellular cytotoxicity (ADCC) *in vivo*. However, since schistosomula are more susceptible to ADCC, it is possible that adult-worm-specific antibody responses may rather target the incoming larvae, a process termed “concomitant immunity” (as predicted by Fisher [88]) and well demonstrated in rhesus monkeys [195].

As initially demonstrated by in vitro studies [196–198], ADCC is dependent on Th2 cytokines, and these have been involved in resistance to schistosome infection. Thus, higher ratios of IL-4/IFN-*γ* and IL-5/IFN-*γ* were produced by specific T-cell clones from *S. mansoni* resistant than susceptible individuals [199] and IL-5 correlated with lower levels of *S. haematobium *infection [99] and *S. mansoni* reinfection after treatment [200]. Furthermore, IL-4, IL-5, and IL-10 levels were associated with resistance posttreatment while IFN-*γ* was associated with susceptibility [201]. However, significantly higher levels of IFN-*γ* against adult worm and cercariae antigens by PBMCs from resistant individuals compared to those from susceptible individuals [202], suggesting that acquired resistance to human schistosomiasis cannot be exclusively classified into a single T helper cell subset.

Cellular immune responses, although involved in resistance, mediate most of schistosome-related pathology [203, 204], which can be divided into acute and chronic diseases based on disease progression. Acute schistosomiasis is a debilitating febrile disease which often occurs in individuals with no experience of infection. It is characterized by high percentage of eosinophilia, which may be reversed by chemotherapy [205], nausea, urticaria, dry cough, and fever [206, 207]. Anatomically, this stage is accompanied by a dissemination of large and destructive granulomas around the eggs [67, 208]. Chronic schistosomiasis is often referred to as a Th2 disease and accounts for most human immunopathologies in endemic areas [77, 204, 208–210]. As infection becomes chronic, schistosome eggs lodge in the liver, gut (*S. mansoni*), or bladder (*S. haematobium*), and the granulomatous response translates into extensive tissue damage and excessive extracellular matrix protein (ECMP) deposition, leading to fibrosis [210].

## 9. Immunological Interaction between Helminth Antigens and Allergens

### 9.1. Helminth Infection and the “Mast Cell Saturation” Hypothesis

The earliest protective mechanism of helminth infection suggested was “mast cell saturation,” whereby helminths induce high levels of nonspecific IgE that saturate Fc receptors on mast cells, thus inhibiting hypersensitivity reactions [211, 212]. Further supportive evidence for the Fc saturation hypothesis came from a study showing that histamine release of human mast cells from lung fragments could be blocked by preexposure of these fragments to high total IgE [213]. However, more recent studies on basophils have shown that high levels of polyclonal IgE and polyclonal/specific IgE ratios from filarial- and hookworm-infected patients do not prevent antigen-induced histamine release [214, 215]. Nevertheless, Mitre and colleagues were able to show that extremely high ratios of polyclonal/specific IgE, enhanced with polyclonal myeloma IgE *in vitro*, could prevent histamine release [214]. Although basophils and mast cells may be differentially regulated [216], these experiments suggested that the Fc*ε*RI receptor saturation may not be the primary mechanism by which helminths “protect” against allergy.

### 9.2. Helminth Infection and Cross-Reactive IgE Responses

Another hypothesis suggested was that helminth parasites induce a “clinically irrelevant” allergen-specific IgE response, which would be cross-reactive between helminths and allergens [217]. Cross-reactive anti-tropomyosin IgE antibodies between helminths and allergens have recently been demonstrated, where monkeys infected with *Loa loa* (filarial parasites) mounted an IgE cross-reacting between filarial tropomyosin and Derp 1 allergen but not with timothy grass [218]. Furthermore, cross-reactivity between ascaris and mites has been reported [219]. However, field studies report mixed results on the effects of helminth infections on allergen-specific IgE in endemic areas [220–222]. Our recent study has demonstrated that the levels of anti-Derp1 IgE antibodies inversely correlate with *S. haematobium* infection intensity in a high schistosome infection area in Zimbabwe [223].

### 9.3. Helminth-Induced Immunomodulation

Technological and scientific advances such as genomic sequences and proteomic approaches have generated molecular and evolutionary information on the relationship between helminth parasites and allergic reactivity. Helminth infections are generally characterised by a Th2-polarised immune response [25, 84, 224], which is often associated with host resistance to infection/reinfection [30]. However, this Th2 response is also associated with pathology [204], consistent with the role for Th2 in allergic diseases [171]. Nevertheless, helminth parasites are capable of modulating this response to prolong their survival and minimize severe pathology in their host [76, 84, 225]. This immunomodulation is thought to affect unrelated antigens such as allergens, hence dampening the clinical manifestation of allergy. Indeed, experimental studies have demonstrated helminth-induced suppression of allergic responses via multiple pathways (Figure 1). However, these observations and mechanisms remain to be rigorously tested in humans. Furthermore, biological and evolutionary differences in the mouse experimental host and the natural human host must be taken into account when extrapolating mechanistic and phenomenological results from the mouse to the human, for example, differences in the IgE receptors [226].

Human studies investigating the regulatory mechanisms underlying the protective effect of helminth infections on atopy have primarily focused on IL-10. Thus, parasite-induced IL-10 production and skin prick reactivity were negatively associated in *Ascaris lumbricoides* [42] and *Schistosoma haematobium* [46] infected populations. Furthermore, allergen-induced IL-10 was associated with reduced Th2 responses (IL-4 and IL-5) in asthmatic schistosome infected patients [227]. More recently, it has been shown that the frequency of PBMCs expressing cytotoxic-T-lymphocyte antigen 4 (CTLA-4) and monocytes expressing IL-10 from asthmatic patients infected with *S. mansoni* was significantly higher compared to their asthmatic uninfected counterparts [228]. However, a study on an Ecuadorian population showed no association between skin prick reactivity with either IL-10 or IL-10-producing T cells induced by *Ascaris lumbricoides* [40].

**Figure 2:**
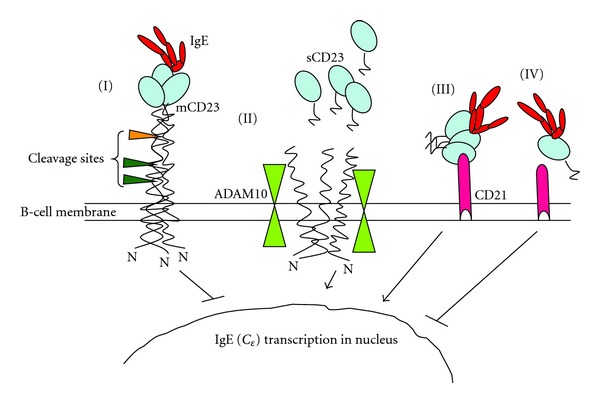
Interaction between CD23 and its ligands, IgE and CD21. Binding of IgE stabilises membrane-bound CD23 and inhibits IgE synthesis (I) from activated B cells while in the absence of IgE binding the CD23 is cleaved by ADAM10 (a disintegrin and metalloprotease protein 10) and this destabilisation enhances IgE synthesis (II). However, soluble CD23 (sCD23) fragments resulting from the cleavage have the ability to bind IgE with different affinities depending on their oligomerization state: trimers (III) bind IgE with high affinity while monomers (IV) bind with low affinity. Trimers enhance IgE synthesis by their ability to also bind CD21 receptor (III) while monomers fail to bind CD21 and inhibit IgE synthesis (IV) (adapted from [32–36]).

The TGF*β* is another cytokine involved in the modulation of immune responses, and is secreted by antigen-presenting cells (APCs) or regulatory T cells [229]. However, there is a paucity of human studies on this cytokine in the context of atopy and helminth infections. Interestingly, we have observed a negative association between atopy and the levels of soluble CD23 in *S. haematobium* infected populations (Rujeni et al., manuscript in preparation). The CD23 is the low affinity receptor for IgE and is involved in the regulation of these antibodies [35]. As illustrated in Figure 2, the soluble CD23 can either upregulate or downregulate IgE synthesis depending on their size and oligomerization state. Of note, expression of this receptor has been associated with resistance to schistosome [194] and *Ascaris* [230] infections in humans, and suppressed airway allergy in helminth-infected mice [231].

As illustrated in Figure 1, immunomodulation during chronic helminth infection is driven by regulatory T and B cells (Tregs and Bregs, resp.), which secrete the above mentioned anti-inflammatory cytokines. Treg cells are either recruited by Bregs or induced and expanded by helminth-derived products [28]. Both T and B regulatory cells can suppress Th2 cells thereby regulating atopy and helminth-induced pathology [31, 232]. Indeed, a study in our lab has shown that Treg proportions correlate with the levels of schistosome infection in young children actively acquiring infection [233].

Helminth molecules have been identified from excretory-secretory (ES) products that are associated with immunomodulation during helminth infection. Thus, the ES-62 is a phosphorylcholine-containing glycoprotein secreted by *Acanthocheilonema viteae*, a rodent filarial nematode [234]. This protein presents anti-inflammatory properties and has been successfully tested in mouse models of allergy and autoimmune diseases [235, 236], and it is currently being exploited as a potential therapeutic agent for inflammatory diseases in humans [237]. The anti-inflammatory properties of this molecule include modulation of B-cell proliferation and cytokine production as well as hyporesponsiveness and desensitization of mast-cell degranulation [237–240]. The interleukin-4-inducing principle from* S. mansoni* egg IPSE/alpha-1, identified as one of the most abundant proteins secreted by *S. mansoni* eggs [241], has also been associated with immunomodulation, possibly by inducing granulomatous responses [242]. Furthermore, IPSE/alpha-1 has been shown to induce antigen-independent IL-4 production by murine basophils in vivo [243]. The venom allergen-like (VAL) proteins are another group of helminth ES products involved in immunomodulation. Thus, Hewitson et al. have demonstrated that antibodies to these VAL antigens are dominant in susceptible mice in an *H. polygyrus* infection model [244]. Sj-VAL-1 is one of the VAL proteins identified in *S. japonicum* egg ES products inducing an antibody response during the first 6 weeks of infection in mice [245].

## 10. Convergence of Allergic and Antiparasite Responses

There is current interest in determining the common features in the induction of immune responses by allergens and by helminths as well as the evolutionary advantages of maintaining allergic responses. As illustrated above, several studies have suggested that similarities in antigens may underlie the commonality of Th2 responses elicited by allergens and by helminths. A recent review [246] of allergic responses indicated that there is relatively little structural similarity between different allergens (e.g., house dust mite, food allergens, and haematophagous fluids) and between allergens and helminth parasites. Instead, this paper suggests that the relationship between allergic and antiparasite Th2 responses arises from a common response to different classes of environmental challenges which include helminth parasites, venoms and haematophagous fluids, and environmental irritants such as carcinogens and noxious xenobiotics, so that this diverse group of stimuli activates responses collectively known as “allergic host defences” [246]. Within this paradigm, these environmental challenges are characterised only by the type of response they elicit with multiple pathways leading to the activation of Th2 responses with the result of protecting against environmental challenges by either reduced exposure to, or elimination of the “irritant.” In this scenario, allergic reactivity is believed to have evolved as an important and essential mechanism against harm rather than a harmful overreaction of a misdirected immune system [247]. Studies in cancer patients also show a negative association between cancer and atopy which has led to the suggestion that allergy protects against some types of cancer [248, 249]. This suggests that the Th2 responses protecting against allergens, carcinogens, and helminths are complex. This presents a challenge for the development of therapeutics relying on helminth products to overcome allergic responses, since induction of allergic responses as well as the effector mechanisms maybe tightly regulated, and the effector responses they elicit may have been selected for redundancy.

## 11. Conclusions

We have shown similarities in the immunological responses to schistosome parasites and to allergens. Studies continue to determine the aetiology of the similar responses and the evolutionary pathways that may have led to the development and maintenance of allergic responses which are paradoxically harmful to the host [246, 247], but may be essential to protect against harm from environmental challenges [246]. The clinical manifestation of atopy is complex with several studies from helminth endemic areas having shown that allergic sensitisation and clinical manifestation of allergy can be dissociated [222]. Furthermore, allergic disease and parasitic infections exist as comorbidities in many patients and are not mutually exclusive [250]. The role of impaired serological allergy diagnosis in parasitized allergy patients as well as under diagnosis in developing countries needs to be addressed to inform future studies. Detailed longitudinal and mechanistic studies relating atopy and clinical disease to schistosome infection and disease in human populations will be valuable to inform on not only the immunological process occurring, but more importantly on clinical management of allergy and schistosomiasis patients.

## Figures and Tables

**Table 1:** Heterogeneity in studies investigating the effect of helminth infection on atopy.

Parasite spp, References	Atopy outcome	Association	Population age
*Ascaris lumbrocoides*			
[37]^1^	Wheeze, SPT	Negative	1–4 years
[38]^2^	IgE, PK	Negative	5–15 years
[39]^1^	SPT, airway responsiveness	Positive	8–18 years
[40]^2^	Allergen-induced Th2 cytokines	None	7–13 years
[41]^2^	SPT, wheeze	None	9 years mean age
[42]^2^	SPT	Negative	6–17 years
	Wheeze, eczema, EIB	None	
*Trichuris trichiura*			
[38]^2^	IgE, PK	Negative	5–15 years
[37]^1^	Wheeze, SPT	None	1–4 years
[43]^#^	SPT	Negative	2–8 years
			
Hookworm			
[37]^1^	Wheeze, SPT	None	1–4 years
[42]^2^	SPT	Negative	6–17 years
	wheeze, eczema, EIB	None	
*Schistosoma mansoni*			
[44]^1^	SPT, IgE	Negative	18 ± 9.7 years
[45]^#^	SPT, asthma symptoms	Negative	15 years mean age
*Schistosoma haematobium*			
[46]^1^	SPT	Negative	5–14 years

Cross-sectional ^1^ and treatment followup ^2^ studies are reported here.

^#^Longitudinal approach but treatment intervention was not the primary objective of the study. SPT: skin prick test; PK: Prausnitz-Kustner passive transfer test, EIB: exercise-induced bronchoconstriction.
